# A bioluminescent and homogeneous assay for monitoring GPCR-mediated cAMP modulation and PDE activity

**DOI:** 10.1038/s41598-024-55038-0

**Published:** 2024-02-23

**Authors:** Dareen Mikheil, Matthew A. Larsen, Kevin Hsiao, Nathan H. Murray, Tim Ugo, Hui Wang, Said A. Goueli

**Affiliations:** 1 Promega Corporation, 2800 Woods Hollow Road, Madison, WI 53711 USA; 2 Promega Biosciences, 277 Granada Drive, San Luis Obispo, CA 93401 USA

**Keywords:** Biochemistry, Biological techniques, Biotechnology, Cell biology, Drug discovery

## Abstract

3′,5′-Cyclic adenosine monophosphate (cAMP), the first identified second messenger, is implicated in diverse cellular processes involving cellular metabolism, cell proliferation and differentiation, apoptosis, and gene expression. cAMP is synthesized by adenylyl cyclase (AC), which converts ATP to cAMP upon activation of G_αs_-protein coupled receptors (GPCRs) in most cases and hydrolyzed by cyclic nucleotide phosphodiesterases (PDEs) to 5′-AMP. Dysregulation of cAMP signaling is implicated in a wide range of pathophysiological conditions such as cardiovascular diseases, neurodegenerative and behavioral disorders, cancers, diabetes, obesity, cataracts, and others. Therefore, cAMP targeted therapies have been and are still undergoing intense investigation for the treatment of these and other diseases. This highlights the need for developing assays to detect and monitor cAMP levels. In this study, we show cAMP Lumit assay as a highly specific homogeneous bioluminescent assay suitable for high throughput screenings with a large assay window and a wide dynamic range for cAMP detection. We believe that this assay will aid and simplify drug discovery screening efforts for cAMP signaling targeted therapies.

## Introduction

3′,5′-Cyclic adenosine monophosphate (cAMP), the first identified second messenger, is synthesized by adenylyl cyclase (AC) which converts ATP to cAMP upon activation of G_αs_-protein coupled receptors (GPCRs) in most cases^1,2^. Cyclic nucleotide phosphodiesterases (PDEs) on the other hand degrade cAMP to 5′-adenosine monophosphate (5′-AMP). The rates of cAMP synthesis by AC and hydrolysis by PDEs determine the level of cAMP in the cell^3^. cAMP, as a second messenger, is essential for signal transduction as it transmits and amplifies signals coming from cell surface receptors^4^. Downstream of cAMP are Protein Kinase A (PKA), the guanine-nucleotide-exchange factor (GEF) EPAC, cyclic nucleotide-gated ion channels and Popeye domain containing proteins as main effectors of cAMP signaling^5–8^. The cAMP signaling pathway is entailed in diverse cellular processes involving cellular metabolism^9–11^, cell proliferation and differentiation^12^, apoptosis^13,14^, and gene expression^15^.

Dysregulation of cAMP signaling has been found to be associated with several pathophysiological conditions such as cardiovascular diseases^16,17^, neurodegenerative and behavioral disorders^18–21^, cancers^22,23^, diabetes^24,25^, obesity^26^, cataracts^27^, and others. The broad spectrum of these conditions and their prevalence worldwide highlights the need for continued efforts in cAMP signaling research for targeted therapies. Several studies have investigated targeting the cAMP/PKA pathway for cancer treatment^28,29^, as well as diabetes^30^, cardiovascular, kidney^31^ and other diseases. Targeting the cAMP pathway can be achieved mainly by cAMP level modulation and PKA inhibition. Some of the FDA-approved drugs work by modulating cAMP levels. An example is liraglutide and exenatide which are GLP1RR (GPCR) agonists used to treat diabetes. They trigger insulin secretion by stimulating β cells to produce cAMP. Also, PDE4 inhibitors have been developed that enhance glucose homeostasis in patients^32^. In the case of acute heart failure, colforsin daropate hydrochloride, an AC5-selective forskolin (FSK) derivate, activates AC5 to induce cAMP production^33^. For patients with COPD experiencing severe air flow limitations, symptoms of chronic bronchitis, and a history of exacerbations roflumilast (a PDE4 inhibitor) is used as an FDA approved drug^34,35^. Another FDA approved PDE4 inhibitor is apremilast used for psoriasis and psoriatic arthritis patients.

A number of assays are available for the detection and measurement of cAMP levels in both biochemical and cellular systems. Many of these assays are homogeneous time resolved fluorescence (HTRF)-based that use cAMP specific antibodies which bind to free cAMP or a fluorescently labeled cAMP conjugate (competitor). Fluorescently labeled cAMP binding to the antibody is then detected either by fluorescence resonance energy transfer (FRET) or enzymatic reactions. In addition, there are two assays from Promega for the detection of cAMP. The first one is the GloSensor cAMP assay which uses a split luciferase that reassembles upon cAMP binding. The downsides for this assay are mainly the need for cell engineering and the inability to run calibration curves using a cAMP standard. The other assay is the cAMP Glo bioluminescent assay; a very simple assay and was designed to address the limitations of the GloSensor assay, but it has a limited dynamic range for cAMP detection. Of these assays, the most commonly used for cAMP detection are the HTRF assays; however, fluorescence-based assays require fluorescence detection capable instruments that might not be readily available in every laboratory. In addition, in high throughput screening experiments, some compounds might have fluorescent properties which would interfere with the assay signal and performance and lead to false positives. Furthermore, the assay suffers from what is called the hook effect when the amount of antibody is limited. Bioluminescent assays on the other hand overcome these problems associated with fluorescent assays^36,37^.

In this report, we show the utilization of the NanoLuc Binary Technology (NanoBiT) system^38^ with a cAMP specific antibody and a cAMP tracer to detect cAMP in biochemical enzymatic as well as cellular assays. The assay is homogeneous and specific with a large assay window and a wide dynamic range for cAMP detection. The assay is suitable for high throughput screening (HTS) and can be run in different screening sizes on any simple luminometer. We believe that this assay will aid in simplifying screening efforts in drug discovery for cAMP signaling targeted therapies.

## Materials and methods

### cAMP-small BiT (SmBiT) tracer synthesis

cAMP-PEG3-SmBiT tracer synthesis methods are provided in detail in the supplementary data section.

### Specificity testing and Z′ factor determination

cAMP (Promega, V6421), 2′3′-cyclic adenosine monophosphate (2′3′-cAMP, Sigma A9376), cyclic guanosine monophosphate (cGMP, Promega V6411), 2′3′-cyclic guanosine monophosphate-adenosine monophosphate (2′3′cGAMP, Cayman Chemical 19887) and 3′3′-cyclic guanosine monophosphate-adenosine monophosphate (3′3′cGAMP, Sigma SML1232) were serially diluted (3-folds) starting at 10uM in 1 × Immunoassay Buffer C (IAB-C, Promega VB115B). 20 µl of the diluted metabolites were added to wells in a 96 well plate (Corning 3912). Antibody mix containing 1 ng Anti-cAMP/5ul (GenScript A01509), 15 nM Tracer (final concentration in the reaction), 3:500 diluted Lumit Anti-Mouse Ab-LgBit (Promega W102B) in 2 × IAB-C was prepared. 20 µl of the antibody mix were added to wells containing metabolites. Plates were mixed on an orbital shaker for 5 min (min) at 400 rpm then incubated for 30 min at room temperature. Nano-Glo Luciferase Assay Substrate (Promega N113C) was prepared by diluting the stock solution 12.5-fold in 1 × IAB-C. 10 µl of diluted substrate were then added to reaction wells. Plates were mixed on an orbital shaker for 3 min at 400 rpm before measuring luminescence signal on a GloMax Discover Microplate Reader (Promega GM3000) within 15 min. Metabolite concentrations were reported as initial concentrations in the 20µL prior to antibody mix addition to the wells.

For competition experiments, a similar protocol was followed except that the initial 20ul of the diluted metabolite in the reaction was divided into 10ul of 2X diluted cAMP and 10ul of 2X diluted competitor metabolite (or buffer for control).

For Z’ factor determination, a similar protocol was followed except that cAMP was diluted to 1uM, 0.5uM, 0.25uM, 0.1uM, 0.05uM and 0.025uM. The assay was run for cAMP at each concentration alongside a buffer control for comparison (48 replicates each, on the same plate). Z’ factor values were calculated according to the previously described method (briefly described below in the statistics methods section).^39^

### PDE4 inhibition study

PDE4A1A enzyme (BPS Biosciences 60040) was diluted to 2 ng/µL in 1XPDE Reaction Buffer (40 mM Tris pH 7.5, 10 mM MgCl2, 0.1 mg/mL BSA). 10µL were added to wells in a 96 well plate. Ro-20–1724 or Difamilast (TargetMol T27172), PDE4 inhibitors, were serially diluted in 1XPDE Reaction buffer keeping dimethyl sulfoxide (DMSO, Sigma D2650) concentration equal between all dilutions. 10µL of diluted inhibitor were added to wells containing the enzyme. Plates were mixed on an orbital shaker for 10 min at 400 rpm. 10µL of 30 µM cAMP (final 10 µM in the 30µL) in 1XPDE Reaction Buffer were added as the substrate needed to initiate the reactions. Plates were mixed on an orbital shaker for 5 min at 400 rpm and then incubated for another 25 min at room temperature. 20µL of 1.25 mM IBMX in 1XPDE Reaction Buffer were added to wells to terminate reactions. Plates were mixed on an orbital shaker for 5 min at 400 rpm. Each well then received 50µL of the antibody mix containing 1 ng Anti-cAMP/5ul, 15 nM Tracer (final concentration in the reaction), 3:500 diluted Lumit Anti-Mouse Ab-LgBit in 2 × IAB-C and plates were mixed for 5 min at 400 rpm and incubated for 30 min at room temperature. Nano-Glo Luciferase Assay Substrate was prepared by diluting the stock solution 12.5-fold in 1 × IAB-C. 25µL of the diluted substrate were then added to each well. Plates were mixed on an orbital shaker for 3 min at 400 rpm. Luminescence signal was detected on a GloMax Discover Microplate Reader within 15 min. Inhibitor concentrations were reported as initial concentrations in the 30µL enzyme reaction before excess IBMX were added to terminate reactions. Percent inhibition was determined by normalizing to a no inhibitor control.

### Cell based assays

HEK293, A375, DRD1 stably transfected HEK293 and DRD2 stably transfected HEK293 cells were maintained in Dulbecco's Modified Eagle Medium (DMEM, Gibco 11995-065) + 10% fetal bovine serum (FBS, Avantor 89510-194) + 1% Penicillin–Streptomycin (Gibco 15140-122). Media used for stably transfected cells was further supplemented with 500ug/mL of Genetecin (Gibco 10131035). Cells were trypsinized (Gibco 25200-056) and counted using an automated cell counter (BioRad TC20). Cells were plated in 384 well plates (Corning 3570) at 1000 cells/well in 25µL media and allowed to adhere and recover overnight at 37 °C/5% CO_2_. The next day, compounds and cAMP standard were serially diluted in Hank's Balanced Salt Solution (HBSS, Gibco 14025-092) containing 500 µM 3-isobutyl-1-methylxanthine (IBMX, Sigma I5879) and 100 µM Ro 20-1724 (Sigma B8279). Media was removed from wells and 5µL of the diluted compounds were added to each well. 5µL of diluted cAMP were added to wells without cells for standard curve. Plates were mixed on an orbital shaker for 5 min at 400 rpm. Treatment incubation (at room temperature) times as follows: 1 h for Dopamine (Sigma H8502), SKF38393 (Sigma D047), SCH23390 (Sigma 505723), Quinpirole (Sigma Q102), L741,626 (Tocris 1003) and NECA (Sigma 119140); 30 min for FSK (Sigma F6886); 5 min for Isoproterenol (Tocris 1747), epinephrin (Sigma 1236970), Propranolol (Sigma P0884) and Alprenolol (Sigma A0360000). 5µL of 2% trichloroacetic acid (TCA, Fisher Scientific SA9410-500) in water were added to wells to terminate reactions and lyse cells. 5µL of 2% TCA were also added to cAMP wells. Plates were mixed on an orbital shaker for 5 min at 400 rpm. 5µL of the antibody mix containing 2 ng Anti-cAMP/5ul, 15 nM Tracer (final concentration in the reaction) and 3:500 diluted Lumit Anti-Mouse Ab-LgBit in 0.25X IAB-C/0.3M Tris, pH 9.0 were added to each well. Plates were mixed on an orbital shaker for 5 min at 400 rpm then incubated for 30 min at room temperature. 5µL of Nano-Glo Luciferase Assay Substrate diluted 26-fold in 0.25 × IAB-C were then added to each well and plates were mixed on an orbital shaker for 3 min at 400 rpm. Luminescence signal was measured on a GloMax Discover Microplate Reader within 15 min. Reported compounds concentrations are concentrations in the initial 5µL treatment before TCA addition, while reported cAMP concentrations are final concentrations in the reaction.

## Software and statistics

For Z’ factor calculations and statistical analyses Excel software was used ($Z^{\prime}=1-\frac{3*({SD}_{s}+{SD}_{c})}{\mid {\mu }_{s}-{\mu }_{c}\mid }$); in this equation *μ* = mean, *SD* = standard deviation, *s* = sample and *c* = control. For standard curves, inhibitor studies and cellular assays statistical calculations and figures generation GraphPad Prism 9.1.0. was used to determine the EC50/IC50 using Nonlinear regression (curve fit) using sigmoidal dose response. In all instances *n* = the number of independent sample replicates within an experiment and *SD* indicates the standard deviation.

## Results and discussion

### Assay principle and format

**Figure 1:**
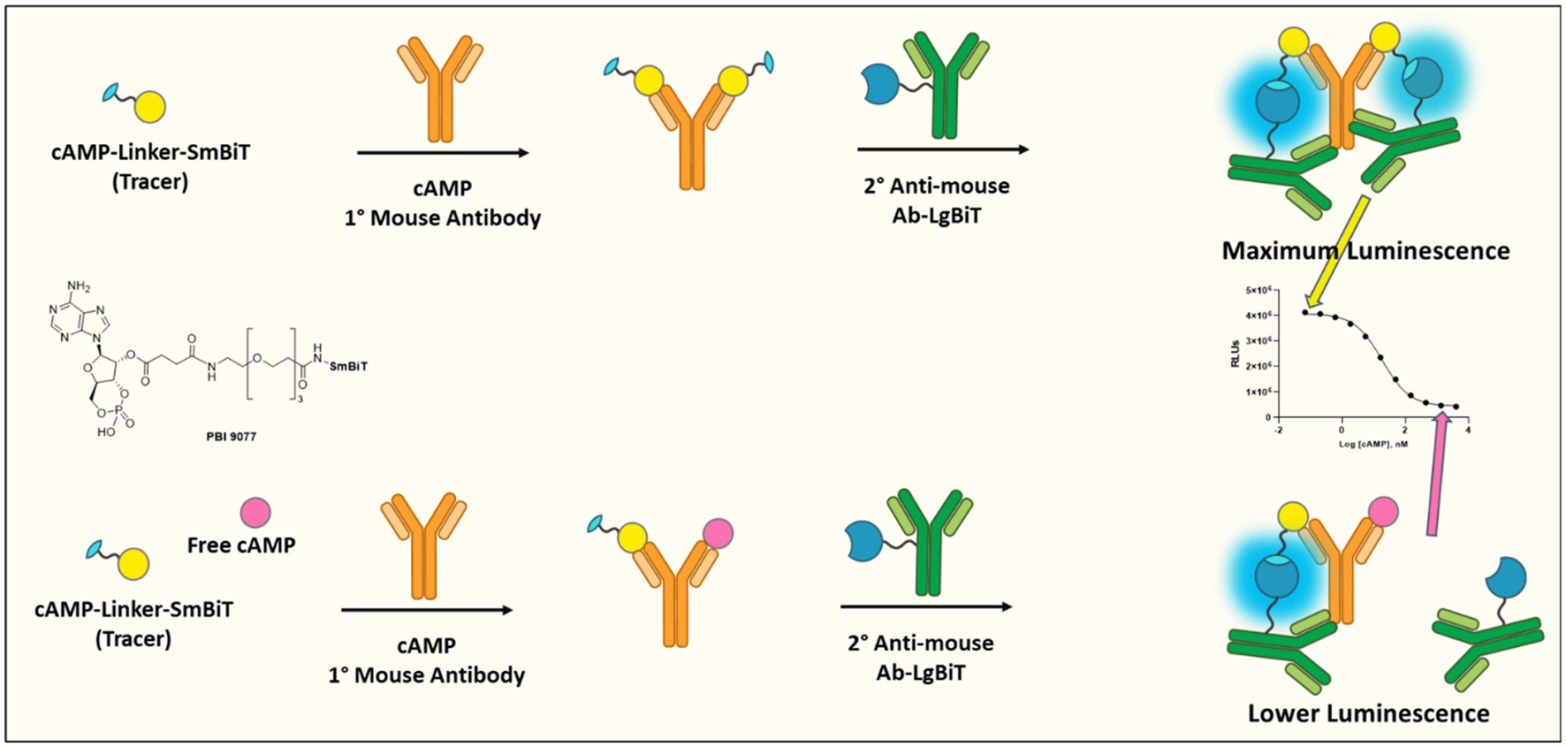
Schematic representation of the cAMP Lumit assay principle.

The cAMP Lumit immunoassay system is a homogeneous competitive assay for detecting and monitoring cAMP in biochemical enzymatic reactions or in cellular systems. Figure 1 shows a schematic representation of the cAMP Lumit assay. Similar to other Lumit technologies^40^, this assay relies on the NanoLuc Binary Technology (NanoBiT)^38^. In a NanoBiT system, a structural complementation between the Large BiT (LgBiT) luciferase subunit and the Small BiT (SmBiT) peptide, when brought into close proximity, results in the formation of a functional enzyme that generates luminescence, in the presence of its substrate^38^, that is proportional to the extent of NanoBiT complementation. In this assay, a cAMP-SmBiT tracer molecule that resembles the endogenous cAMP is employed along with a cAMP specific primary antibody and a secondary antibody conjugated to the LgBiT subunit. If there is no cAMP present in the reaction, the cAMP-SmBiT tracer complements the LgBiT subunit forming functional luciferase enzyme which produces light in the presence of its substrate. On the other hand, if there is cAMP in the reaction, it binds to the antibody competing with the cAMP-SmBiT tracer. This results in a lower level of NanoBiT complementation that is manifested in a lower bioluminescence signal.

### Assay development

**Figure 2:**
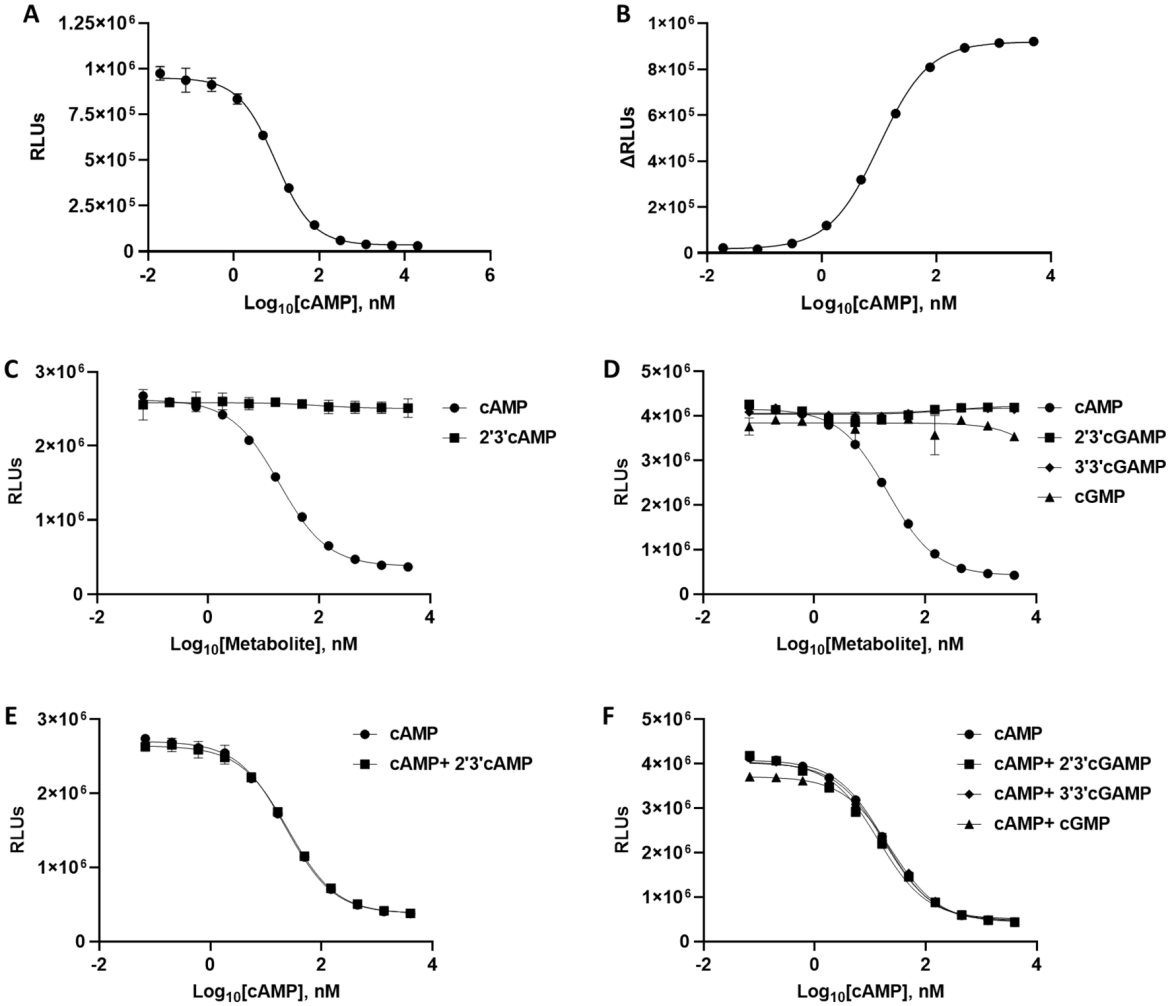
cAMP Lumit Assay range and specificity. (**A**,**B**) cAMP standard curve (n = 3 ± SD). (**C**,**D**) Titration of other metabolites in comparison with cAMP titration in cAMP Lumit assay (n = 2 ± SD). (**E**,**F**) 10 µM of potential interfering metabolites (or buffer) were included in a cAMP titration assay (n = 2 ± SD).

To build the cAMP Lumit assay, a functional antibody-tracer pair was necessary. We found the combination of an anti-cAMP primary antibody from GenScript (A01509) with a cAMP-SmBiT tracer containing a PEG3 linker between the cAMP and the SmBiT peptide showed the highest performance. In the presence of 10 µM cAMP (final concentration), this combination consistently resulted in more than 25-fold lower signal compared to the no cAMP control (Assay window). Figure 2A shows a dose dependent response to a cAMP standard (EC_50_ = 9.6 nM cAMP). The assay range in this example was determined using the EC_10_ = 0.85 nM of cAMP and the EC_90_ = 106.9 nM of cAMP (EC_10_–EC_90_ = 106.1 nM of cAMP). Figure 2B shows the same cAMP dose response data (shown in Fig. 2A) by plotting ΔRLUs against cAMP concentration. Figure 2C,D show the cAMP Lumit assay specificity. This was tested by titrating another form of cAMP (2′3′-cAMP) or potential cross-reactive metabolites (2′3′-cGAMP; 3′3′-cGAMP; cGMP) in the system, instead of cAMP. None of the tested metabolites resulted in a significant luminescence signal drop at concentrations up to 10 µM. To test for the interference ability of these molecules with the assay, they were included in the assay at a concentration of 10 µM. Nearly identical cAMP standard curves were generated showing no effect on the cAMP Lumit assay’s ability to detect cAMP as shown in Fig. 2E,F. Hence, we concluded that the assay is specific, and that other cellular metabolites should not hinder its cAMP detection ability.

**Figure 3:**
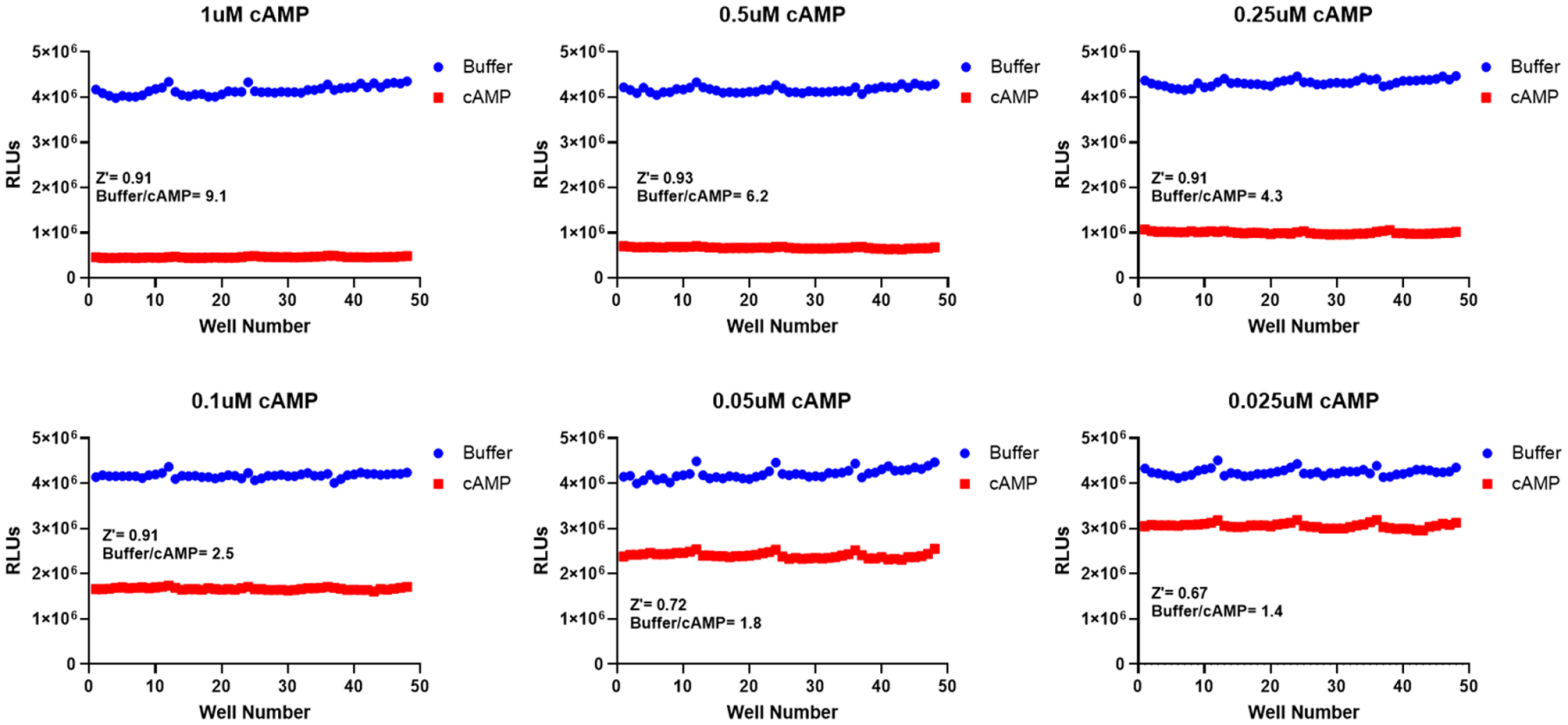
Z′ factor test for the cAMP Lumit assay. cAMP Lumit assay was run for the indicated cAMP concentrations and compared to buffer controls to assess assay performance and calculate Z′ factors at each concentration.

To determine the robustness and reproducibility of the cAMP Lumit assay, Z′ factor values were calculated for 1, 0.5, 0.25, 0.1, 0.05 and 0.025 µM of cAMP. A Z′ factor of 0.5 or more (maximum 1) is interpreted as an excellent assay for high throughput screening. The luminescent signal in wells containing each of the mentioned cAMP concentrations was compared to buffer containing wells in a 96 well format (Fig. 3). For all the tested concentrations the Z’ factor values were 0.67 or more. This data shows that the cAMP Lumit assay is both robust and reproducible which makes it suitable for high throughput screening.

### Measurement of PDE inhibition

**Figure 4:**
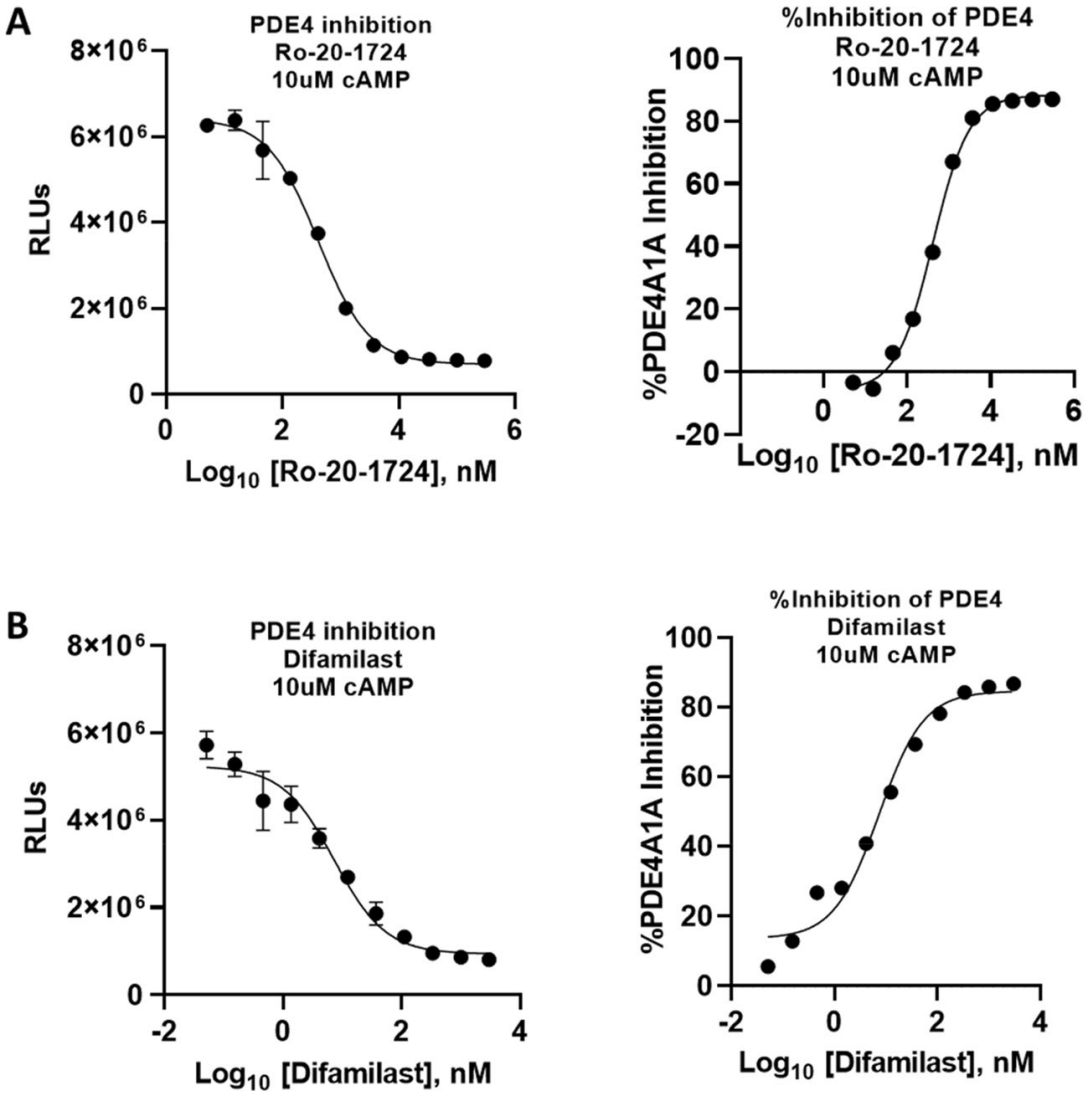
cAMP Lumit assay detects PDE inhibition. (**A**) Ro 20-1724, and (**B**) Difamilast. The right side of each figure shows the transformation of the data into percent inhibition of the data on the left side. Inhibitor titrations were carried out using 20 ng PDE4A1A per reaction in the presence of 10 µM cAMP (*n* = 2 ± SD).

PDEs constitute the main path through which cAMP is hydrolyzed. To date, 11 PDE families have been characterized. One way for PDE classification is substrate specificity. While PDE4, PDE7, and PDE8 are cAMP specific, PDE5, PDE6 and PDE9 are cGMP specific. On the other hand, PDE1, PDE2, PDE3, PDE10, and PDE 11 use both cAMP and cGMP as substrates^41,42^. Three PDE4 inhibitors, apremilast, crisaborole, and roflumilast, are FDA approved and they are used for skin and lung diseases treatment^43^ demonstrating the promise of targeting cAMP signaling. To show the value of our assay in PDE inhibition studies and targeting for drug discovery, we tested the PDE 4 inhibitors Ro 20–1724 and Difamilast in the cAMP Lumit assay. Our results showed that both Ro 20–1724 and Difamilast inhibit PDE4A1A in a dose dependent manner with IC_50_ = 416.3 nM and 7.3 nM, respectively (Fig. 4). This biochemical study indicates the suitability of the cAMP Lumit assay in monitoring PDE activity, which is of great value in drug discovery and development.

### cAMP detection in cellular systems

As mentioned above, cAMP signaling mostly gets initiated through GPCRs, which constitute the largest protein family and are the most common target of FDA approved drugs. To date, about 134 GPCRs are targeted by approved drugs (more than 700 drugs), however none of these GPCRs are orphan receptors, which highlights the high potential for more discoveries and further studies in this area. Moreover, novels ways for targeting GPCRs continue to be under investigation aiming to generate different classes of drugs such as biased signaling drugs, allosteric modulators, antibodies, aptamers, gene therapy agents, and others. As such, the targeting of GPCRs and cAMP signaling will remain a hot area of research for drug discovery^44^.

**Figure 5:**
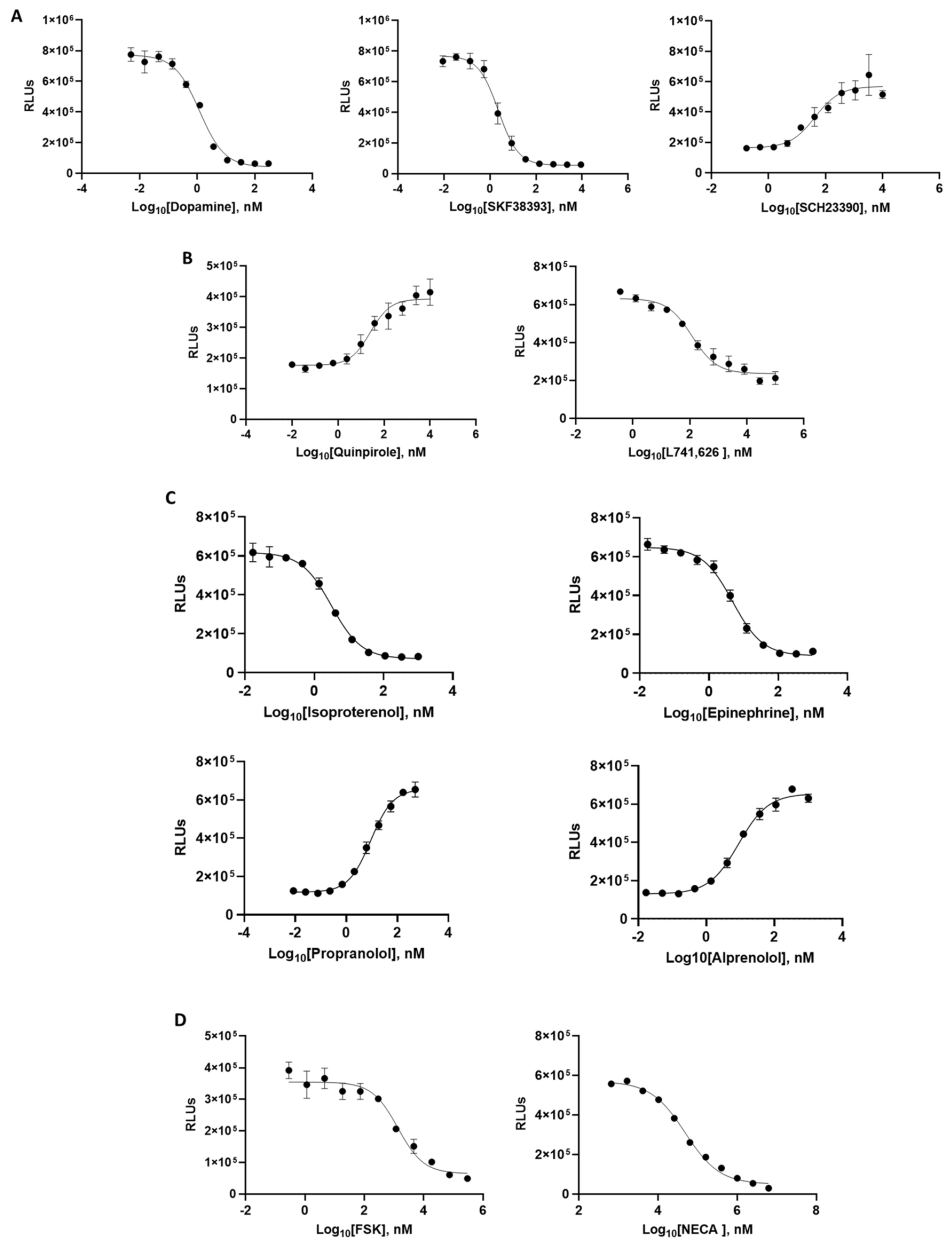
cAMP detection in cellular systems. (**A**) HEK293 cells stably transfected with DRD1 were treated with the DRD1 agonists Dopamine or SKF38393 leading to cAMP levels increase, or the DRD1 antagonist SCH23390 (in the presence of 100 nM SKF38393) leading to cAMP levels reduction (*n* = 3 ± SD). (**B**) HEK293 cells stably transfected with DRD2 were treated with the DRD2 agonist Quinpirole (in the presence of 5 µM FSK) leading to cAMP levels reduction, or the DRD2 antagonist L741, 626 (in the presence of 200 nM Quinpirole + 8 µM FSK) leading to cAMP levels increase (*n* = 3 ± SD). (**C**) A375 cells were treated with the β-Adrenergic agonists Isopreterenol or Epinephrine leading to cAMP levels increase, or the β-Adrenergic receptor antagonsits Propranolol or Alprenolol (in the presence of 50 nM Isopreterenol) leading to cAMP levels reduction (*n* = 3 ± SD). (**D**) HEK293 cells were treated with Forskolin and A375 cells were treated with the Adenosine receptor agonist NECA, both leading to cAMP levels increase (*n* = 3 ± SD).

To demonstrate the applicability of cAMP Lumit in cellular assays, we manipulated cAMP levels in different cell lines using different GPCR agonists and antagonists (Table 1) and employed cAMP Lumit for the detection of cAMP levels changes in these cells. The assays were conducted by treatment of cells followed by lysis with 2% TCA, which also terminates the reactions. Cell lysates were then incubated with antibodies and tracer diluted in 0.3 M Tris buffer (pH 9.0; for neutralization) for 30 min after which time the detection reagent was added. All treatments resulted in a detectable change in cAMP levels by our cAMP Lumit Assay (Fig. 5). Even with the effect of TCA and Tris treatment on the assay background, reduction of luminescent signal ranged between 2.2 and 20-fold, maintaining a good assay window for cAMP detection in cellular systems. Taken together, the data presented here show that the cAMP Lumit assay is a competent assay that can be easily adapted for the detection of cAMP production in different cellular systems for the support of GPCR and cAMP-related drug discovery.

**Table 1:** GPCR agonists and antagonists for cAMP levels manipulation.

Receptor	Gα-submit	Cell line	Agonist	Antagonist	Figure
Dopamine D1	Gαs	HEK293 cells stably transfected with DRD1	Dopamine; SKF38393	SCH23390 in the presence of 100 nM SKF38	5A
Dopamine D2	Gαi	HEK293 cells stably transfected with DRD2	Quinpirole in the presence of 5uM FSK	L741, 626 in the presence of 200 nM Quinpirole + 8 um FSK	5B
β-Adrenergic	Gαs	A375 cells, endogenous receptor levels	Isopreterenol; Epinephrine	Propranolol; Alprenolol in the presence of 50 nM isopreterenol	5C
Adenosine	Gαs	A375 cells, endogenous receptor levels	NECA	**	5D
**	**	HEK293	FSK	**	5D

## Conclusion

cAMP signaling is key for various cellular processes and it has been implicated in several diseases. The targeting of cAMP signaling for drug discovery is anticipated to continue to grow, which mandates the availability of a simple and reliable cAMP detection system. Commercially available cAMP detection assays either require cell engineering or need specialized plate readers capable of fluorescent signal detection or are ELISA-based. The cAMP Lumit assay demonstrated in this study, on the other hand is a simple, sensitive, specific, and direct assay that requires no cell engineering, which can get laborious and limiting for biosensor-based assays with some types of cells. In addition, cAMP Lumit has a broader dynamic range in comparison with other available assays, only needs a simple luminometer and is not susceptible to interference with fluorescent compounds. Moreover, the assay is suitable for HTS and automation, a feature that ELISA-based assays lack. Our data show the assay’s robustness and competence in both enzymatic biochemical and cellular assays which makes it the perfect choice for drug screening for cAMP signaling targeted therapies.

### Supplementary Information

Supplementary Information.
