# Supplementary material for: A bioluminescent and homogeneous assay for monitoring GPCR-mediated cAMP modulation and PDE activity
Source: Sci Rep. 2024 Feb 23;14:4440. doi: 10.1038/s41598-024-55038-0 (PMC10891162; doi:10.1038/s41598-024-55038-0)
Supplement: Supplementary file 1 — Supplementary Information. [file 41598_2024_55038_MOESM1_ESM.docx]

Supplementary Data

General Experimental Details for Chemical Synthesis

SmBiT peptide (VTGYRLFEEIL) was purchased from GenScript or synthesized in-house on a peptide synthesizer (CSBio Instrumentation). All other reagents and solvents for chemical syntheses were purchased from Aldrich, TCI America, Combi-blocks, and Fisher, and were used without further purification. Purifications of synthetic intermediates and final SmBiT-cAMP product were performed on a Waters Preparation HPLC (Waters 2487 Series) using 0.1% aqueous trifluoroacetic acid and acetonitrile as eluents. Reaction progress monitoring was performed using a Waters LC-MS with a Waters 2695 Separation Module/3100 Mass Detector. The purity and confirmation of identity of the key cAMP NHS ester product was determined by ^1^H NMR, ^13^C NMR, and HRMS. The purity and confirmation of identity for peptide intermediates and final SmBiT-cAMP products were determined by LC-MS and HRMS. HRMS mass spectra were collected via Sciex TripleTOF® 5600+. NMR spectra were collected with a 400 MHz Bruker NMR. All purified products were lyophilized from MeCN/water.

Synthesis of SmBiT-PEG3-NH_2_

To a 20 mL vial was added SmBiT (30.0 mg, 0.0224 mmol), BocNH-PEG3-NHS (14.1 mg, 0.0336 mmol), DIPEA (0.010 mL, 0.056 mmol), anhydrous DMSO (2 mL). The mixture was stirred at room temperature for 1 h. To the mixture was added TFA (5 mL) and water (1 mL). The mixture was stirred for an additional 2 h. The mixture was purified by reversed phase HPLC and lyophilized to afford SmBiT-PEG3-NH_2_ as a colorless fluffy solid (27.8 mg, 0.0180 mmol, 80% yield). HRMS/ESI [M+H]^+^ calc’d for C_72_H_116_N_16_O_21_ 1541.8574, found 1541.8603.

Synthesis of cAMP-NHS

To a 20 mL vial was added cAMP (25.0 mg, 0.0794 mmol) and water (3 mL). To the mixture was added Et_3_N (0.052 mL, 0.38 mmol). A solution formed. To the mixture was added succinic anhydride (9.5 mg, 0.095 mmol) as a solution in THF (2 mL), dropwise. After 15 min, the mixture was concentrated to dryness. The residue was co-evaporated with dry MeCN 3 times to remove residual water. The residue was dissolved in DMF (1.5 mL). To the mixture was added TSTU (29.7 mg, 0.0988 mmol) and DIPEA (0.027 mL, 0.152 mmol). The mixture was stirred at room temperature. After 15 min, the mixture was acidified with AcOH (0.1 mL) and diluted with water (4 mL). The mixture was purified by reversed phase HPLC and lyophilized to afford cAMP-NHS (12.0 mg, 0.0187 mmol, 25% yield). ^1^H NMR (400 MHz, DMSO-*d*_6_) δ 8.46 (s, 1H), 8.34 (s, 1H), 8.24 (s, 2H), 6.28 (s, 1H), 5.79 (d, *J* = 5.7 Hz, 1H), 5.25 (ddd, *J* = 9.5, 5.7, 1.9 Hz, 1H), 4.51 (ddd, *J* = 21.3, 8.7, 4.1 Hz, 1H), 4.20 (dtd, *J* = 14.7, 9.9, 6.5 Hz, 2H), 3.01 (t, *J* = 6.6 Hz, 2H), 2.87 (t, *J* = 6.3 Hz, 1H), 2.81 (s, 4H), 2.70 (s, 2H).

^13^C NMR (101 MHz, DMSO) δ 170.8, 170.6, 168.6, 158.9, 158.5, 148.9, 142.0, 120.0, 89.3, 76.1, 73.4, 71.9, 68.0, 28.6, 26.3, 25.9.

HRMS/ESI [M-H]^-^ calc’d for C_18_H_19_N_6_O_11_P 525.0777, found 525.0768.

^1^H NMR spectrum

^13^C NMR spectrum

Synthesis of cAMP-PEG3-SmBiT

To a 20 mL vial was added cAMP-NHS (10.0 mg, 0.0190 mmol), SmBiT-PEG3-NH_2_ (29.3 mg, 0.0190 mmol), DMF (1 mL), and DIPEA (0.010 mL, 0.057 mmol). The mixture was stirred at room temperature. After 1 h, the mixture was purified by reversed phase HPLC and lyophilized to afford cAMP-PEG3-SmBiT (2.2 mg, 0.0011 mmol, 6% yield). HRMS/ESI [M+2H]^2+^ calc’d for C_86_H_130_N_21_O_29_P 976.9613, found 976.9634.

The datasets used and/or analysed during the current study available from the corresponding author on reasonable request.
